# Clinical Analysis of 32 Cases of Subglottic Benign Airway Stenosis Treated With Montgomery T Silicone Stent

**DOI:** 10.1155/2024/2145560

**Published:** 2024-10-16

**Authors:** Zhenyu Yang, Xiaoli Zhou, Wenying Pan, Daxiong Zeng, Junhong Jiang

**Affiliations:** ^1^The Fourth Affiliated Hospital of Soochow University (Suzhou Dushu Lake Hospital), Department of Respiratory and Critical Care Medicine, Suzhou 215000, Jiangsu, China; ^2^The First Affiliated Hospital of Soochow University, Department of Respiratory and Critical Care Medicine, Suzhou 215000, Jiangsu, China

**Keywords:** complication, long-term follow-up, montgomery T silicone stent, stent removal, subglottic benign airway stenosis

## Abstract

**Objective:** To explore the complications of long-term placement of Montgomery T silicone stent (T*-*tube) in the treatment of subglottic benign airway stenosis (SBAS) and the timing of successful T*-*tube removal.

**Methods:** We retrospectively collected the clinical data of 32 patients with SBAS who underwent the treatment of T*-*tube and analyzed their placement and successful removal of the T*-*tube.

**Results:** There were 22 males and 10 females, aged from 21 to 79 years (60.9 ± 13.7 years). The T*-*tubes were successfully placed in all 32 patients, and 6 patients (18.8%) with mild stenosis were placed by the intravenous conscious sedation. The longest follow-up period was 60.4 months, and 17 patients (53.1%) had the T*-*tubes for more than 12 months; 5 patients (15.6%) were changed to the tracheostomy cannula after unplanned removal of the T*-*tubes for various reasons; the T*-*tubes were successfully removed in 9 patients (28.1%), and the duration of T*-*tubes placement was 5.2–22.7 months (12.1 ± 6.3 months), among them anatomical stenosis in 9 patients (100%). Secretion retention was observed in 32 patients (100%), granulation tissue hyperplasia was observed in 9 patients (28.1%), and the normal ventilation was not affected in most patients by bronchoscopic treatment and follow-up; the T*-*tubes were removed in 3 patients due to severe complications. There was no significant difference in the incidences of secretion retention and granulation tissue hyperplasia between the time point at 1 week, 1 month, 3 months, and 12 months, *p* > 0.05. In patients with T*-*tube more than 12 months, the severity of secretion retention at 1 week, 1 month, 3 months, and 12 months was significantly different, *p* < 0.05, however, there was no significant difference in the severity of granulation tissue hyperplasia, *p* > 0.05.

**Conclusions:** T*-*tube is safe and effective in the treatment of SBAS. The severity of secretion retention increased in patients with long-term placement of the T*-*tube. For patients with mild stenosis and anatomical stenosis, the T*-*tube removal can be attempted at about 1 year of follow-up.

## 1. Introduction

SBAS refers to the narrowing of the airway from the inferior border of the vocal cord to the inferior border of the cricoid cartilage caused by various benign etiologies, with acquired stenosis being more common; the primary cause of acquired SBAS is iatrogenic injury, such as tracheostomy or endotracheal intubation [1]. Laryngotracheal resection and airway reconstruction is the preferred treatment for SBAS, however, the clinical application is limited by the high demands on cardiopulmonary function and the high incidence of surgical complications [2, 3]. With the continuous development of respiratory intervention techniques, the T*-*tube has been considered an effective and safe treatment for SBAS. However, due to the difficulty of T*-*tube placement and the high requirements for anesthesia techniques, there is currently a lack of the large-scale clinical studies and the data of long-term follow-up on T*-*tube treatment for SBAS, and there is no consensus on the indications for the removal of T*-*tube [4–6]. We retrospectively analyzed the clinical data of 32 patients of SBAS treated with the T*-*tubes, compared the occurrence of complications at different time points during long-term follow-up, and attempted to remove the T*-*tubes in appropriate patients, aiming to provide reference for the clinical treatment of such patients.

## 2. Materials and Methods

### 2.1. Patients

This study was approved by the Ethics Committee of the Fourth Affiliated Hospital of Soochow University (Suzhou Dushu Lake Hospital); the informed consent was signed in all patients in the study.

A retrospective analysis was conducted on the clinical data of 32 patients with SBAS who underwent the T*-*tubes treatment at the Fourth Affiliated Hospital of Soochow University and the First Affiliated Hospital of Soochow University from May 2016 to November 2023. There were 22 males and 10 females, with ages ranging from 21 to 79 years (average age: 60.9 ± 13.7 years). The causes of SBAS were tracheostomy in 25 patients (78.1%), endotracheal intubation in 1 patient (3.1%), tracheal trauma in 2 patients (6.3%), and laryngeal surgery in 4 patients (12.5%). The primary diseases leading to SBAS were cerebral hemorrhage in 14 patients (43.7%), cerebral infarction in 2 patients (6.3%), cervical trauma in 2 patients (6.3%), severe pneumonia in 3 patients (9.4%), spinal surgery in 2 patients (6.3%), brain surgery in 1 patient (3.1%), Hashimoto's encephalopathy in 1 patient (3.1%), Guillain-Barré syndrome in 1 patient (3.1%), aortic dissection in 1 patient (3.1%), tetanus in 1 patient (3.1%), and laryngeal surgery in 4 patients (12.5%). The patients in this study generally had a poor underlying condition due to the long-term treatment of the primary disease, so the T*-*tube was selected as the treatment for SBAS rather than the surgery (Table 1).

### 2.2. Preoperative Assessment and Treatment Before T*-*Tube Placement

All 32 patients with SBAS in this study met the indications for the T*-*tube placement, including patients with severe stenosis, patients with mild stenosis who relapsed after treatment with other methods, and patients with dynamic airway stenosis [7, 8]. In 25 patients with tracheotomy, the tracheotomy cannula could not be removed, and the extubation difficulty occurred after the primary diseases were stabilized. And recurrent stenosis after routine interventional bronchoscopy therapy (including high-frequency electric knife, argon gas, laser, and cryotherapy) occurred in these 25 patients with SBAS due to tracheotomy. Recurrent stenosis after balloon dilation and metal stent occurred in 1 patient with SBAS due to endotracheal intubation. Two patients with tracheal trauma who underwent tracheal intubation and tracheotomy had recurrent stenosis and extubation difficulty after receiving the routine interventional therapy of airway. In 4 patients with SBAS due to laryngeal surgery, repeated stenosis was still occurred after the routine interventional therapy of airway (Table 1).

**Figure 1:**
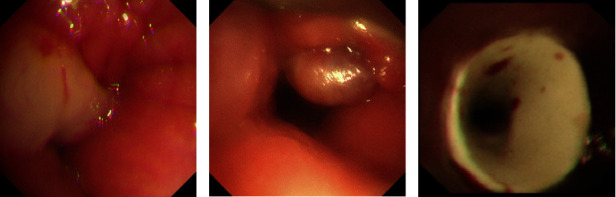
Typical subglottic benign airway stenosis and T*-*tube placement. (a) Hypertrophic mucosa above the tracheotomy cannula, (b) proliferating granulation tissue above the tracheotomy cannula, and (c) successful placement of T*-*tube.

Prior to the T*-*tube placement, all 32 patients underwent chest computed tomography (CT) scanning, three-dimensional reconstruction, and bronchoscopy. (1) There are 2 types of airway stenosis [9]: anatomical stenosis in 29 patients (90.6%), dynamic stenosis in 3 patients (9.4%); among patients with anatomical stenosis, the type stenosis of intraluminal growth in 24 patients (Figures 1(a) and 1(b)), the stenosis of mixed type in 5 patients. (2) The severity of stenosis was assessed according to the Cotton-Myer grading system [10]: Grade I in 5 patients (15.6%), Grade II in 11 patients (34.4%), Grade III in 8 patients (25.0%), and Grade IV in 8 patients (25.0%). Grades I and II were considered mild stenosis, while Grades III and IV were considered severe stenosis. (3) Before the T*-*tube placement, 19 cases (59.4%) underwent endoscopic interventions, including high-frequency electric knife, argon gas, laser, and cryotherapy in 18 patients, balloon dilation in 5 patients, and metal stent placement in 2 patients (Table 1).

### 2.3. T*-*Tube Placement

The conventional method for T*-*tube placement typically requires the general anesthesia [4, 7]. The methods of T*-*tube placement include: (1) The T*-*tube was placed through the site of tracheotomy by placing a rigid tracheoscope under general anesthesia and connecting to the high-frequency ventilation; (2) The T*-*tube was placed through the site of tracheotomy by placing a laryngeal mask under general anesthesia and ventilation with the ventilator [4, 11]. (3) In our study, the T*-*tube placement under intravenous conscious sedation was also attempted.

### 2.4. Observation and Follow-Up After T*-*Tube Placement

Aerosol therapy (budesonide and acetylcysteine) was given for the patients at least 3 times/day after the placement of T*-*tubes or discharge from hospital to promote the discharge of secretion. When patients' clinical symptoms stabilized, the bronchoscopy follow-up was performed routinely at 1 week, 1 month, and 3 months after T*-*tube placement, and followed by bronchoscopy reexamination every 2-3 months or longer intervals based on clinical symptoms thereafter.

There is no uniform standard for judging the severity of secretion retention; the following scoring criteria were adopted in our study based on clinical experience: (1) score and grade for secretion retention: 0 point for no secretion retention; one point for a small amount of secretion adherent to the wall or both ends of the T*-*tube, easily removed under bronchoscopy; two points for the secretion adherent to the wall or both ends of the T*-*tube, blocking less than 1/3 of the lumen but difficult to remove under bronchoscopy; three points for more viscous secretion adherent to the wall or both ends of the T*-*tube, or blocking more than 1/3 of the lumen and difficult to remove under bronchoscopy; four points for secretion retention leading to the removal of the T*-*tube. (2) Score and grade for granulation tissue hyperplasia [12]: 0 point for no granulation tissue hyperplasia; one point for the granulation tissue hyperplasia at both ends of the T*-*tube narrowed the lumen diameter by less than 25%; two points for the granulation tissue hyperplasia at both ends of the T*-*tube narrowed the lumen diameter by 25%–50%; three points for the granulation tissue hyperplasia at both ends of the T*-*tube narrowed the lumen diameter by less than 50%–75%; four points for the granulation tissue hyperplasia at both ends of the T*-*tube narrowed the lumen diameter by more than 75%; five points for granulation tissue hyperplasia leading to the removal of T*-*tube. (3) As for the secretion retention, grades 0, 1, and 2 were classified as mild complications, and grades 3 and 4 were classified as severe complications. As for the granulation tissue hyperplasia, grades 0, 1, and 2 were classified as mild complications, and grades 3, 4, and 5 were classified as severe complications.

### 2.5. Statistical Analysis

Data were analyzed by using SPSS 27.0 statistical software. The occurrence of complications was described using the median and interquartile range. Fisher's exact test or Friedman's test was used for comparing complications at different time points, with *p* < 0.05 considered statistically significant.

## 3. Results

### 3.1. T*-*Tube Placement

T*-*tubes (Boston Medical Products, Inc.) were successfully placed in all 32 patients (Figure 1(c)). Three sizes of T*-*tube were selected in our study, 11 millimeter (mm) in 13 patients (40.6%), 12 mm in 18 patients (56.3%), and 13 mm in 1 patient (3.1%). 23 patients (71.9%) underwent the T*-*tubes placement under general anesthesia with the rigid bronchoscope, including 10 patients with mild stenosis and 13 patients with severe stenosis; 5 patients (15.6%) with mild stenosis underwent T*-*tubes placement under intravenous conscious sedation with the laryngeal mask and controlled ventilation; 1 patient (3.1%) with mild stenosis underwent the T*-*tube placement under intravenous conscious sedation with nasal high-flow oxygen therapy; 3 patients (9.4%) with severe stenosis underwent the T*-*tubes placement under general anesthesia with tracheostomy and the support of ventilator (Table 1).

### 3.2. Overall Complications After T*-*Tube Placement

**Figure 2:**
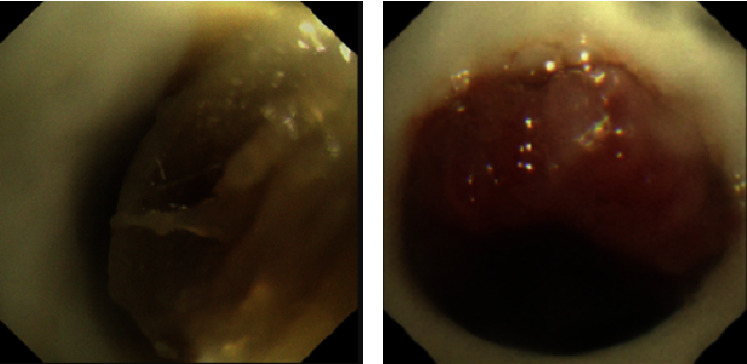
Typical manifestations of complications. (a) Phlegm scab in the middle of T*-*tube (severe complication) and (b) proliferating granulation tissue in the lower end of T*-*tube (mild complication).

All 32 patients (100%) experienced the complications of varying degrees after the T*-*tube placement, mainly secretion retention or granulation tissue hyperplasia, with severe complications in 6 patients (18.8%) (Figure 2(a)) and mild complications in 26 patients (81.2%) (Figure 2(b)). Secretion retention was observed in all 32 patients (100%), with no impact on normal ventilation after regular bronchoscopic cleaning and home nebulization treatment in 29 patients. Nine patients (28.1%) developed granulation tissue hyperplasia, of whom 8 patients experienced resolution or no increase in granulation tissue after bronchoscopic treatment or follow-up. Additionally, the T*-*tubes were removed due to severe secretion retention in 2 patients, and the T*-*tube was removed due to severe secretion retention and granulation tissue in 1 patient.

### 3.3. Occurrence of Complications at Different Time Points

1. Occurrence of secretion retention at different time points: (i) At 1 week after the T*-*tube placement, the incidence of secretion retention was 93.8% (30/32). (ii) At 1 month after the T*-*tube placement, the incidence of secretion retention was 100% (31/31). (iii) At 3 months after the T*-*tube placement, the incidence of secretion retention was 100% (28/28). (iv) At 12 months after the T*-*tube placement, the incidence of secretion retention was 100% (17/17). There were no significant differences in the incidence of secretion retention at 1 week, 1 month, 3 months, and 12 months after the T*-*tube placement (*p* > 0.05) (Table 2).
2. Occurrence of granulation tissue hyperplasia at different time points: (i) At 1 week after the T*-*tube placement, the incidence of granulation tissue hyperplasia was 15.6% (5/32). (ii) At 1 month after the T*-*tube placement, the incidence of granulation tissue hyperplasia was 12.9% (4/31). (iii) At 3 months after the T*-*tube placement, the incidence of granulation tissue hyperplasia was 14.3% (4/28). (iv) At 12 months after the T*-*tube placement, the incidence of granulation tissue hyperplasia was 11.8% (2/17). There were no significant differences in the incidence of granulation tissue hyperplasia at 1 week, 1 month, 3 months, and 12 months after the T*-*tube placement (*p* > 0.05) (Table 2).

### 3.4. Prognosis and Outcome

**Figure 3:**
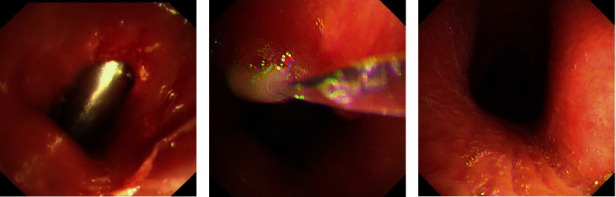
Successful removal of T*-*tube. (a) Successful removal of T*-*tube and placement of metal tracheotomy cannula, (b) a little secretion in the above of tracheotomy cannula after successful removal of T*-*tube at 1 week, and (c) no obvious secretions and granulation tissue in the above of tracheotomy cannula after successful removal of T*-*tube at 1 week, and successful removal of metal tracheotomy cannula in this patient in the end.

As of February 22, 2024, the 32 patients were followed up for 1 week to 60.4 months. Unplanned removal of T*-*tubes occurred in 5 patients (15.6%), including 2 patients due to severe secretion retention, 1 patient due to severe secretion retention and granulation tissue hyperplasia, 1 patient due to glottic dysfunction, and 1 patient due to accidental removal by the patient himself. 18 patients (56.3%) were still carrying the T*-*tubes at the cut*-*off point of follow-up time or death. The T*-*tubes were successfully removed in 9 patients (28.1%) (Figures 3(a), 3(b), and 3(c)), with follow-up durations ranging from 5.2 to 22.7 months and an average follow-up duration of 12.1 ± 6.3 months. In the patients who had the T*-*tubes successfully removed, anatomical stenosis in 9 patients (100%), mild stenosis in 6 patients (66.7%), various forms of interventional treatment before the T*-*tube placement in 6 patients (66.7%).

Among the 17 patients with long-term placement of T*-*tubes (follow-up for 12 months), the scores for secretion retention were as follows: 1 (1, 1) point at 1 week after the T*-*tube placement, 1 (1, 1) point at 1 month after the T*-*tube placement, 1 (1, 1) point at 3 months after the T*-*tube placement, and 2 (2, 2) points at 12 months after the T*-*tube placement. There was a significant difference in scores of secretion retention between 12 months and 1 week, 1 month, and 3 months after the T*-*tube placement (*p* < 0.05) (Table 3). The scores for granulation tissue hyperplasia were as follows: 0 (0, 0) point at 1 week after the T*-*tube placement, 0 (0, 0) point at 1 month after the T*-*tube placement, 0 (0, 0) point at 3 months after the T*-*tube placement, and 0 (0, 0) point at 12 months after the T*-*tube placement. There was no significant difference in scores of granulation tissue hyperplasia at different time points (*p* > 0.05) (Table 3).

## 4. Discussion

Benign airway stenosis is one of the common diseases of the respiratory system, which is a type of disease caused by pathological changes in the wall of airway due to various etiologies. Its clinical symptoms vary depending on the severity of the stenosis. Mild stenosis may present as coughing, chest tightness, while severe stenosis may manifest as difficulty breathing, respiratory failure, or even death. The treatment of benign airway stenosis is challenging due to different etiologies, locations, and degrees of stenosis [13, 14]. SBAS refers to stenosis occurring in the region between the inferior border of the vocal cord and the inferior border of the cricoid cartilage caused by benign diseases. The most common causes of SBAS are endotracheal intubation and tracheostomy [7]. Stenosis caused by endotracheal intubation is due to balloon compression, and the risk factors include prolonged intubation, especially exceeding 7 days, while stenosis caused by tracheostomy is similar to tracheal injury, resulting from granulation tissue hyperplasia and cartilage injury due to abnormal wound healing [9]. In our study, 26 patients with SBAS (81.3%) were caused by endotracheal intubation or tracheostomy.

 The surgery used to be the preferred treatment for SBAS. However, the poor surgical tolerance due to poor underlying condition in some patients, the high incidence of surgical complications and the recurrence rates limit its clinical application to a certain extent [2, 3]. In recent years, with the continuous development of interventional pulmonology, patients' clinical symptoms can be effectively improved by the methods of treatment including high-frequency electric knife, laser, cryotherapy, and balloon dilation [15]. However, the stent placement may be a better option for patients with dynamic airway stenosis. The placement of metal stent is relatively simple and can rapidly improve malignant airway stenosis, but it is not recommended for the treatment of benign airway stenosis due to its complications [16, 17]. In our study, different forms of bronchoscopic intervention therapy were performed on 19 patients (59.4%) before the T*-*tube placement but the recrudescent stenosis was occurred in above patients.

Silicone stents are more widely used in the treatment of benign airway stenosis due to their better tissue compatibility and fewer complications [18]. The T*-*tube is a type of silicone stent that can be placed and removed through the site of tracheotomy. Rare migration was occurred in the T*-*tube and easier sputum aspiration can be completed because of the presence of the outer branch. And vocal function can be restored and the quality of life can be significantly improved by closing the outer branch. Therefore, the T*-*tube placement is a good choice for SBAS caused by tracheostomy [7]. In the study by Wu et al. [5] and others, 25 patients with airway stenosis (13 of whom were caused by endotracheal intubation or tracheostomy) underwent the T*-*tubes placement in the observation group, while 27 patients with airway stenosis (15 of whom were caused by endotracheal intubation or tracheostomy) underwent the therapy of balloon dilation in the control group; the results showed that compared with the control group, the stenosis and clinical symptoms weresignificantly improved at 1 week after the T*-*tubes placement in the observation group. In our study, the success rate of placement of T*-*tubes was 100%. The T*-*tube was removed due to severe secretion retention only in 1 patient at 1 week, and the degree of stenosis and clinical symptoms were improved in the remaining patients at 1 week after the T*-*tube placement.

General anesthesia combined with rigid bronchoscopy is the conventional method for the T*-*tube placement. Proficiency in rigid bronchoscopy is required for the T*-*tube placement, and the position of hyperextension of the neck should be maintain during the operation [7]. However, rigid bronchoscopy may not be completed if the patients with spinal cord injuries. Additionally, a series of pulmonary complications such as infection, atelectasis, pulmonary edema, and respiratory insufficiency can be caused by the general anesthesia. Pre-existing stenosis of airway may be aggravated by these complications [19], thus the application of general anesthesia in the intervention treatment of airway stenosis may be limited. Intravenous conscious sedation is a form of anesthesia in which narcotic drugs are administered intravenously to achieve mild suppression of consciousness while allowing the patient to respond to external stimuli, such as speech and light touch; the tolerance and comfort of patients can be improved and the risk of general anesthesia can be reduced by using this method of anesthesia [20, 21]. Jeyabalan and Medford [21] pointed out in their study that the diagnostic efficacy of endobronchial needle aspiration under ultrasound guidance can be ensured but also the satisfaction with the surgical process can be enhanced through the intravenous conscious sedation. In our study, 6 patients (18.8%) with mild stenosis underwent successful placement of the T*-*tube by using intravenous conscious sedation. Among them, 5 patients were ventilated by laryngeal mask combined with anesthesia machine and placed the T*-*tubes through the site of tracheotomy, while 1 patient with spinal cord injury underwent the placement of the T*-*tube via the site of tracheotomy under nasal high-flow oxygen therapy with guidance from soft bronchoscopy. This suggests that intravenous conscious sedation can be a new option for the placement of the T*-*tube, but further research is needed to explore its application in different patient populations and particularly in patients with severe subglottic or airway stenosis.

The main complications after the T*-*tube placement are secretion retention and granulation tissue hyperplasia, with migration being rare [22]. In the study by Hu et al. [23], 20 patients with subglottic and tracheal stenosis resulting from tracheotomy underwent the treatment of T*-*tubes, with a postoperative incidence of secretion retention of 100% and granulation tissue hyperplasia of 65%. In our study, the incidence of secretion retention after the T*-*tube placement was also 100%, while the incidence of granulation tissue hyperplasia was (28.1%). And most patients remained normal ventilation after the treatment during follow-up, while the T*-*tubes were removed due to severe complications in 3 patients (9.4%). However, the short*-*term outcomes or the overall incidence of complications was mainly focused on the current research on complications of the T*-*tube treatment for the subglottic and tracheal stenosis but lacking the comparisons of complications at different time points [5, 23]. In our previous animal experiments, different types of complications (secretion retention, granulation tissue hyperplasia, and scar contraction) occurring at different time points after stent placement in rabbit trachea [24]. In this study, secretion retention and granulation tissue hyperplasia were the main complications, and there was no significant difference in complications at different time points (1 week, 1 month, 3 months, and 12 months) after the T*-*tube placement. These results suggest that the occurrence of complications at different time points during long-term T*-*tube placement for the subglottic and tracheal stenosis can be controlled, indicating better tissue compatibility in the T*-*tube. However, in 17 patients who underwent long-term T*-*tube placement (follow-up exceeding 12 months), secretion retention was significantly more severe at 12 months after the T*-*tube placement compared to 1 week, 1 month, and 3 months. This may be due to the prolongation of follow-up time or the reduction in frequency, indicating the need for enhanced the education of patients and regular bronchial follow-up to avoid the occurrence of severe complications.

Although the long-term placement of the T*-*tube is safe and well-tolerated, the removal of the T*-*tube to further improve quality of life was desired in some patients. The life-long stent placement is necessary for dynamic SBAS, which results from cartilage destruction and loss of airway support. However, there remains controversy in the optimal duration of the T*-*tube for patients of anatomical stenosis [23, 25]. In studies by Carretta et al. [26] and others, 9 patients of SBAS were successfully removed the T*-*tubes and it was suggested that the removal of the T*-*tube could be attempted after 18 months of the placement, however, details regarding the type and severity of stenosis in patients with successful removal of the T*-*tubes were not discussed. In another study by Jin et al. [4], the removal of T*-*tubes was attempted in 9 patients of SBAS at a follow-up of 18–24 months and 5 patients (55.6%) were successfully removed, the failure of removal in 4 patients with severe stenosis (Grade III or IV). In our study, the removal of the T*-*tube was successfully removed in 9 patients (28.1%) with anatomical stenosis, with a follow-up period ranging from 5.2 to 22.7 months (average 12.1 months). And the mild stenosis in 6 of these patients (66.7%). This suggests that for patients with mild, anatomical stenosis, the removal of T*-*tube could be attempted after approximately 1 year of follow-up.

The T*-*tube for SBAS is safe and effective, but its conventional placement requires general anesthesia and rigid bronchoscopy, which demand high basic situation of the patient and proficiency of operator. For patients with the mild stenosis or certain special cases, the intravenous conscious sedation offers a new direction for the T*-*tube placement. However, further research is needed to determine the appropriate patient population and the effects of this anesthesia method on operation time and postoperative complications. Complications at different time points after the T*-*tube placement are controllable and similar, but for patients with long-term T*-*tube placement, regular bronchoscopic follow-up is essential to avoid severe complications. It may be feasible to remove the T*-*tube after approximately 1 year for patients with mild, anatomical stenosis, but more data are required to support this practice. In conclusion, the timing of removal of the T*-*tube should be based on individual factors, including stenosis type, severity, and length.

## Figures and Tables

**Table 1:** Clinical baseline characteristics of the patients.

Variables	*N* = 32
Age	60.9 ± 13.7 (21–79)
Gender (M/F)	22/10
Causes of SBAS (%)	
Tracheotomy	25 (78.1%)
Tracheal intubation	1 (3.1%)
Tracheal trauma	2 (6.3%)
Laryngeal surgery	4 (12.5%)
Types of stenosis (%)	
Anatomical stenosis	29 (90.6%)
Dynamic stenosis	3 (9.4%)
Severity of the stenosis (%)	
Cotton-Myer I	5 (15.6%)
Cotton-Myer II	11 (34.4%)
Cotton-Myer III	8 (25.0%)
Cotton-Myer IV	8 (25.0%)
Interventional therapy before T*-*tube (%)	
Yes	19 (59.4%)
None	13 (40.6%)
Method of T*-*tube placement (%)	
General anesthesia + rigid tracheoscope	23 (71.9%)
Intravenous conscious sedation + laryngeal mask	5 (15.6%)
Intravenous conscious sedation + soft bronchoscope	1 (3.1%)
General anesthesia + tracheotomy	3 (9.4%)

**Table 2:** Comparison of complications at different time points after T*-*tube placement.

Point of time	Complications	
	Secretion retention	Granulation tissue hyperplasia
1 week (*N* = 32)	93.8% (30/32)	15.6% (5/32)
1 month (*N* = 31)	100% (31/31)	12.9% (4/31)
3 months (*N* = 28)	100% (28/28)	14.3% (4/28)
12 months (*N* = 17)	100% (17/17)	11.8% (2/17)
—	*p*=0.34	*p*=1.00

**Table 3:** Scores for the occurrence of complications in patients with long-term T*-*tube placement (follow-up for more than 12 months).

Point of time	Complications	
	Secretion retention (point) (*N* = 17)	Granulation tissue hyperplasia (point) (*N* = 17)
1 week	1 (1.1)	0 (0.0)
1 month	1 (1.1)	0 (0.0)
3 months	1 (1.1)	0 (0.0)
12 months	2 (2.2)	0 (0.0)
—	*p* < 0.001	*p*=0.22

## Data Availability

The data that support the findings of this study are available from the corresponding author upon reasonable request.
